# The PI3K/Akt/mTOR pathway as a preventive target in melanoma brain metastasis

**DOI:** 10.1093/neuonc/noab159

**Published:** 2022-02-01

**Authors:** Cedric Tehranian, Laura Fankhauser, Patrick N. Harter, Colin D.H. Ratcliffe, Pia S. Zeiner, Julia M. Messmer, Dirk C. Hoffmann, Katharina Frey, Dana Westphal, Michael W. Ronellenfitsch, Erik Sahai, Wolfgang Wick, Matthia A. Karreman, Frank Winkler

**Affiliations:** Clinical Cooperation Unit Neurooncology, German Cancer Consortium (DKTK), German Cancer Research Center (DKFZ), Heidelberg, Germany; Edinger Institute, Institute of Neurology, University of Frankfurt am Main, Frankfurt am Main, Germany; German Cancer Research Center DKFZ Heidelberg, Germany and German Cancer Consortium DKTK partner site, Frankfurt/Mainz; Frankfurt Cancer Institute (FCI), Frankfurt am Main, Germany; Tumour Cell Biology Laboratory, The Francis Crick Institute, London, UK; Edinger Institute, Institute of Neurology, University of Frankfurt am Main, Frankfurt am Main, Germany; Germany, Senckenberg Institute of Neurooncology, University of Frankfurt am Main, Frankfurt am Main, Germany; Frankfurt Cancer Institute (FCI), Frankfurt am Main, Germany; Clinical Cooperation Unit Neurooncology, German Cancer Consortium (DKTK), German Cancer Research Center (DKFZ), Heidelberg, Germany; Faculty of Biosciences, Heidelberg University, Heidelberg, Germany; Clinical Cooperation Unit Neurooncology, German Cancer Consortium (DKTK), German Cancer Research Center (DKFZ), Heidelberg, Germany; Faculty of Biosciences, Heidelberg University, Heidelberg, Germany; Clinical Cooperation Unit Neurooncology, German Cancer Consortium (DKTK), German Cancer Research Center (DKFZ), Heidelberg, Germany; Neurology Clinic and National Center for Tumor Diseases, University Hospital Heidelberg, INF 400, 69120 Heidelberg, Germany; Department of Dermatology, Medical Faculty and University Hospital Carl Gustav Carus, TU Dresden, Dresden, Germany; Germany, Senckenberg Institute of Neurooncology, University of Frankfurt am Main, Frankfurt am Main, Germany; Frankfurt Cancer Institute (FCI), Frankfurt am Main, Germany; Tumour Cell Biology Laboratory, The Francis Crick Institute, London, UK; Clinical Cooperation Unit Neurooncology, German Cancer Consortium (DKTK), German Cancer Research Center (DKFZ), Heidelberg, Germany; Neurology Clinic and National Center for Tumor Diseases, University Hospital Heidelberg, INF 400, 69120 Heidelberg, Germany; Clinical Cooperation Unit Neurooncology, German Cancer Consortium (DKTK), German Cancer Research Center (DKFZ), Heidelberg, Germany; Neurology Clinic and National Center for Tumor Diseases, University Hospital Heidelberg, INF 400, 69120 Heidelberg, Germany; These authors jointly supervised this work; Clinical Cooperation Unit Neurooncology, German Cancer Consortium (DKTK), German Cancer Research Center (DKFZ), Heidelberg, Germany; Neurology Clinic and National Center for Tumor Diseases, University Hospital Heidelberg, INF 400, 69120 Heidelberg, Germany; These authors jointly supervised this work

**Keywords:** brain metastasis, PI3K/Akt/mTOR pathway, tertiary prevention, dual PI3K/mTOR inhibition, extravasation

## Abstract

**Background:**

Brain metastases (BM) are a frequent complication of malignant melanoma (MM), with limited treatment options and poor survival. Prevention of BM could be more effective and better tolerated than treating established BM in various conditions.

**Methods:**

To investigate the temporo-spatial dynamics of PI3K/Akt/mTOR (PAM) pathway activation during BM formation and the preventive potential of its inhibition, *in vivo* molecular imaging with an Akt biosensor was performed, and long-term intravital multiphoton microscopy through a chronic cranial window in mice.

**Results:**

*In vivo* molecular imaging revealed invariable PAM pathway activation during the earliest steps of brain colonization. In order to perform a long-term intravascular arrest and to extravasate, circulating MM cells needed to activate their PAM pathway during this process. However, the PAM pathway was quite heterogeneously activated in established human brain metastases, and its inhibition with the brain-penetrant PAM inhibitor GNE-317 resulted in only modest therapeutic effects in mice. In contrast, giving GNE-317 in preventive schedules that included very low doses effectively reduced growth rate and number of BM in two MM mouse models over time, and led to an overall survival benefit. Longitudinal intravital multiphoton microscopy found that the first, rate-limiting steps of BM formation - permanent intravascular arrest, extravasation, and initial perivascular growth - are most vulnerable to dual PI3K/mTOR inhibition.

**Conclusion:**

These findings establish a key role of PAM pathway activation for critical steps of early metastatic brain colonization and reveal its pharmacological inhibition as a potent avenue to prevent the formation of clinically relevant BM.

## Introduction

Brain metastases (BM) develop in up to 50% of patients with advanced melanoma, with relevant negative impact on overall survival and quality of life^1^. Despite successes in systemic treatment of MM patients with new immunotherapies and targeted therapies, the intracranial response of patients suffering from BM is still limited, with a progression free survival of about 4 months and a median overall survival of 5,6 – 17,4 months^2–5^. Hence, there is a great need for the development of new therapeutic strategies for treatment or, even better, prevention of BM^1,6^.

The PI3K/Akt/mTOR (PAM) pathway was found to be upregulated in BM when compared to extracranial metastasis and primary tumors^7–9^. Activation of PAM pathway in tumors of many entities is associated with cell proliferation, disease progression, angiogenesis, treatment resistance, and invasion^10^. Therapeutic treatment with single or dual PI3K/mTOR inhibitors has previously shown to reduce the growth of established BM in preclinical studies^11–13^, but failed to lead to intracranial response in MM patients with established BM in a clinical trial^14^.

Treatment of established BM by approved therapies is often accompanied with relevant toxicity^15^, demonstrating the need for a strategy to prevent BM in high-risk patients, optimally with a long-term, well-tolerated targeted therapy^1^. For eventual outgrowth to brain macrometastasis, cancer cells have to master several steps, with earliest ones being the most rate-limiting^16^. In principle, the decisive early appearing steps of brain colonization could be more vulnerable to targeted therapy, and therefore might be inhibited with a lower dose that would not lead to similar clinically relevant effects on established BM^1,6^. Understanding the relevance of PAM pathway regulation during the metastatic cascade could pave the way for the development of a preventive strategy for PAM inhibiting drugs.

Here, our intravital multiphoton microscopy approach was used to perform *in vivo* molecular imaging on a single cell level to elucidate the temporo-spatial dynamics of PAM pathway activation during BM formation. We demonstrate that early critical and rate-limiting events of BM cascade depend on PAM pathway activation, and its inhibition by lower doses led to significantly better antitumor effects than treating established BM with higher doses.

## Material and Methods

For additional Material and Methods, see Supplementary Information.

### Cell lines

Six different models of human melanoma BM were used; 4 primary cell lines isolated from human melanoma BM, H1Dl2^17^, and brain-passaged A2058 cells^18^. For *in vivo* imaging cells expressing green fluorescent protein (GFP) or red fluorescent protein (RFP) were used^12^. A2058 cells were transduced with F01-Clover Akt biosensor^19^ or LeGO-it2-luc2-ires-tdTomato (a kind gift from Jonas Schwickert and Andreas Trumpp).

### Assessment of PAM pathway activation *in vitro*

Melanoma cells were treated with 0.1/1/5 μM GNE-317, Pictilisib or same volume of dimethyl sulfoxide (DMSO) and stained for phospho-Akt or Annexin V. Flow cytometry data were recorded on a FACS Canto II (BD Biosciences) and analyzed using FlowJo version 10 (FlowJo LLC, USA). For Western Blot, A2058 cells were treated for 10 h with 30 μM GNE-317, 10 μM pictilisib or DMSO. Protein expression of Akt, S6RP, mTOR, and their phosphorylated forms was analyzed.

### Immunohistochemistry

Detection of full protein RPS6, 4EBP1, PRAS40 and their phosphorylated forms were automatically performed as similar as described previously^20^ (adjustments outlined in supplementary material) on 3 μm thick slides from FFPE tissue micro arrays. The study was endorsed by the local ethical committee of the Goethe University Frankfurt, Germany (GS 4/09; SNO_01-12).

### Proliferation assays *in vitro*

Melanoma cells were treated with different doses of GNE-317 or Pictisilib, and were monitored in an xCELLigence Real-Time Cell Analyzer (RTCA) system (Roche Diagnostics) as described before^12^.

### Intravital microscopy

NOD scid gamma mice (NSG, >8 weeks, >20 g, inhouse bred) were anesthetized with ketamine/xylazine and a chronic cranial window was implanted as described before^16^. At least 3 weeks later, melanoma tumor cells were suspended in phosphate buffered saline and injected into the left cardiac ventricle^16^. *In vivo* imaging was performed using a ZEISS LSM 7MP equipped with a Coherent Chameleon Ultra II laser, as previously described^16^. All animal procedures were performed according to the German Cancer Research Center’s guidelines and were approved by the Regierungspraesidium Karlsruhe, Germany (governmental authority for laboratory animal research)

### GNE-317 treatment *in vivo*

NSG mice with previously implanted chronic cranial window received GNE-317 (Genentech, 2.5/12.5/25 mg/kg bodyweight, in 0.5% methylcellulose/0.2% polysorbate (MCT)) via oral gavage in a preventive schedule from day 4 to day 28 or in a treatment schedule from day 21 to day 28. Control mice received similar volume of MCT from day 4 to day 28.

### Akt biosensor studies

A2058 melanoma cells transduced with Akt biosensor were treated with 30μM GNE-317, 10 μM pictilisib or DMSO in medium for 10h, followed by live cell *in vitro* imaging. Cytoplasm/nucleus ratio of GFP was calculated, low Akt activity was defined as a ratio below 1 standard deviation (SD) of control cells with high Akt activity. For *in vivo* studies, low Akt activity was confirmed via fluorescence microscopy. Intravital microscopy was performed <120 minutes after injection to assess PAM pathway activation status in intravascular A2058 cells. Additionally, single tumor cells were fate-tracked on 8 timepoints over the course of 28 days enabling to monitor each step of the metastatic cascade and to measure PAM pathway activation status in space and time. High Akt activity was defined as a higher intensity of GFP signal in the cytoplasm relative to nuclear GFP signal^19,20^.

### GNE-317 treatment and blood glucose measurements

Mice received GNE-317 (Genentech, 2.5/12.5/25 mg/kg bodyweight, in 0.5% methylcellulose/0.2% polysorbate (MCT)) or only MCT via oral gavage during period of drug administration. Blood glucose levels were monitored an hour after drug application on two consecutive days, with the point-of-care glucometer Akku-Check Instant (Roche)^21^.

### Whole-body imaging and survival study

Mice were treated either with 2.5 mg/kg/day GNE-317 (preventive schedule) or MCT (control) between day 4-25 following injection with A2058 expressing Luc2, or with 12.5 mg/kg/day GNE-317 from day 25 onwards (treatment schedule). *In vivo* bioluminescence imaging (IVIS Lumina Series III Imaging system, PerkinElmer) was performed in week 3, 4 and 5.

### Data analysis

For quantification of tumor volume or number of cell nuclei images were transferred to Imaris (Bitplane). Statistical analysis was performed using Prism (Version 9.0.1 (128), GraphPad Prism). Statistical significance was stated for P values < 0.05. Different statistical tests were used as indicated in the figure legends. Normal distribution was determined by a Shapiro-Wilk test.

### Illustrations

Figure 2B, 4C and 5A were created with Biorender.com.

## Results

### An Akt biosensor to measure the dynamics of PAM pathway regulation

**Figure 1:**
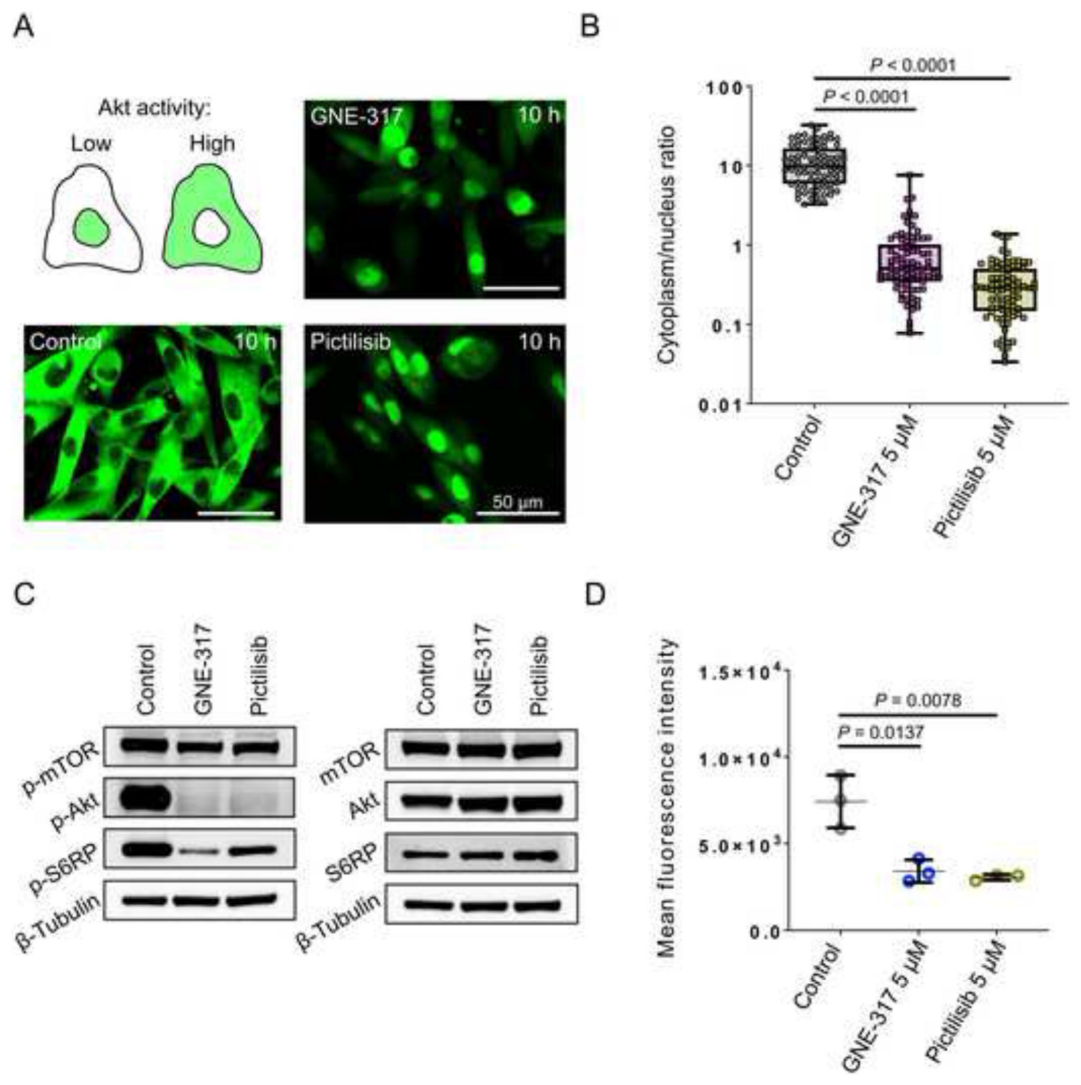
Establishment and validation of an Akt biosensor to measure PAM pathway activation. (A) A2058 melanoma cells expressing the Akt biosensor (green) were seeded in matrigel and incubated with dual PI3K/mTOR inhibitor GNE-317 or selective PI3K inhibitor pictisilib (GDC-0941) for 10 hours. Representative live cell confocal microscopy images of A2058 melanoma cells are shown, cytoplasmic signal represents high Akt activity, nuclear signal represents low Akt activity^19^. (B) Signal intensity was measured in cytoplasm and nucleus, and ratio was calculated. A2058 melanoma cells, n=81, Mann-Whitney test. (C) Western blot analysis of protein levels of p-mTOR(S2448), p-Akt(S473), p-S6RP(S235/236), mTOR, Akt and S6RP in A2058 cells treated with 30 μM GNE-317, 10 μM pictilisib or same amount of DMSO (control) for 10h. ß-Tubulin was used as a loading control. (D) Flow cytometry analysis of p-Akt(S473) expression in A2058 cells treated with 5 μM GNE-317, 5 μM pictilisib or same amount of DMSO (control) for 10h. Mean fluorescent intensity (median), n=3 independent experiments, student’s t-test, error bars show SD.

To gain a thorough understanding of the role of the PAM pathway during BM formation, we aimed to monitor PAM pathway activation in tumor cells at every subsequent step of the brain metastatic cascade *in vivo* and in real time. Hereto, brain metastatic A2058 melanoma cells were transduced with a green fluorescent Akt biosensor which rapidly translocates from the nucleus to the cytoplasm in case of Akt stimulation^19^. First, the Akt biosensor was validated *in vitro.* Proliferating cells showed a cytoplasmic signal of the sensor, indicating high Akt activity, whereas cells that were challenged with a brain penetrant dual PI3K/mTOR inhibitor (GNE-317)^22^ or a selective pan-class I PI3K inhibitor (pictilisib) showed nuclear signal indicating low Akt activity (Figure 1A). To quantify Akt activity, cytoplasm/nucleus ratio of GFP signal was calculated, which confirmed a robust difference (Figure 1B). Accordingly, tumor cells showed lower levels of phosphorylated(p)-S6RP, p-Akt and p-mTOR when treated with GNE-317 or pictilisib (Figure 1C). Likewise, in GNE-317- and pictilisib-treated cells, less p-Akt (Ser473) was detected by flow cytometry (Figure 1D and Supplementary Figure 1).

### Circulating tumor cells rapidly activate the PAM pathway during early brain colonization

**Figure 2:**
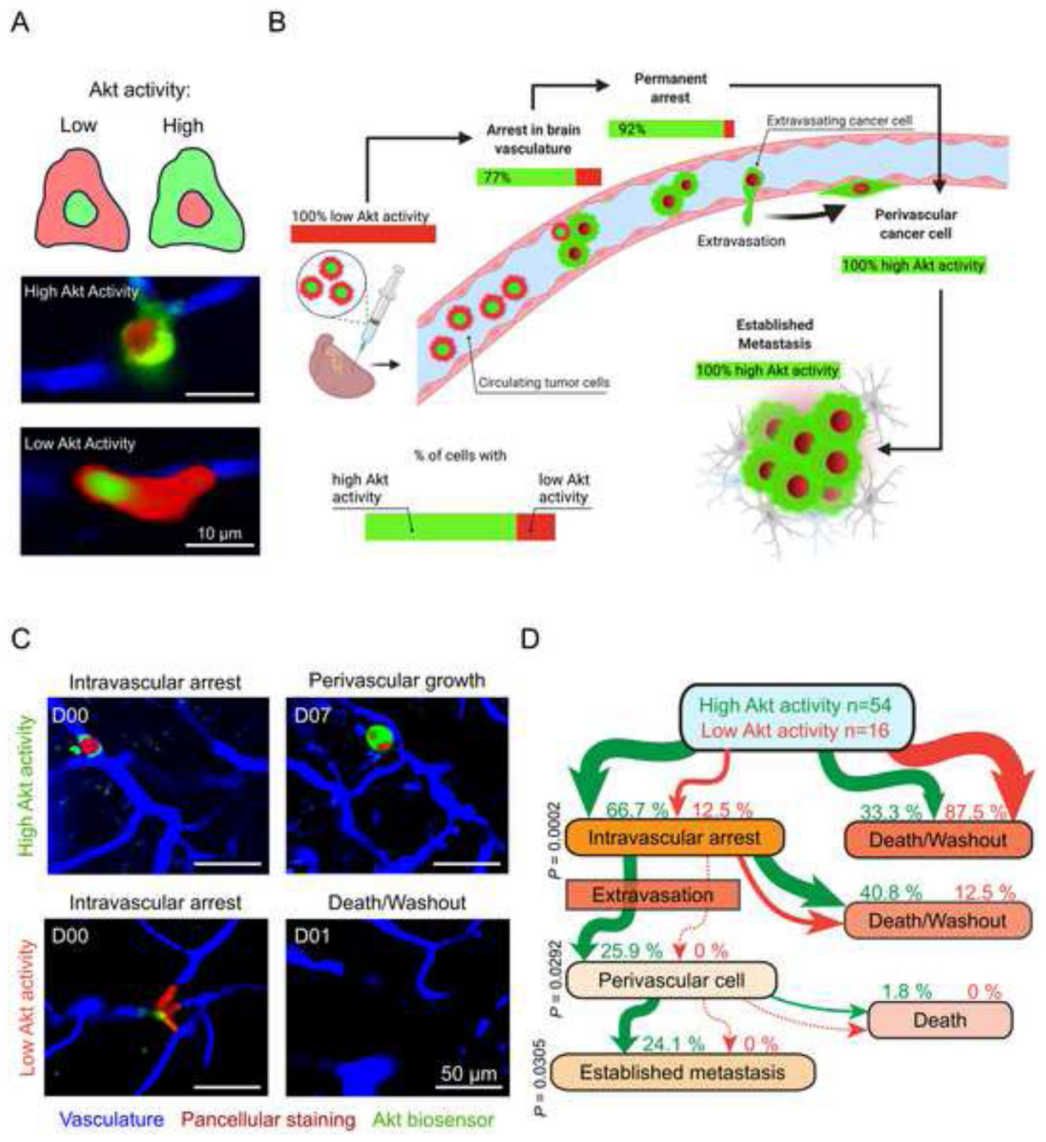
PAM pathway activation is an invariable early event in melanoma BM. (A) Intracardiac injection of biosensor labeled A2058 melanoma cells in mice for longitudinal intravital multiphoton microscopy. *In vivo* measurements of Akt activity on a single cell level in the brain. A2058 melanoma cells, green: Akt biosensor, red: pancellular RFP, blue: brain vessels. Scheme visualizes how to interpret fluorescent signal^19^. (B) Longitudinal intravital multiphoton microscopy; correlation between tumor cell’s PAM pathway activation and the steps of brain metastatic cascade is shown. A2058 cells, n=70 tumor cells in n=5 mice. (C, D) Within first two hours following intracardiac injection, tumor cells can be divided in groups with high or low Akt activity. (C) Representative image shows the need of initial high Akt activity to later colonize the brain. A2058 cells, n=70 in n=5 mice, intravital multiphoton microscopy. (D) Flowchart demonstrates development of BM depending on initial PAM pathway activation. Percentages refer to initially high vs low Akt activity cells in intravascular stage. A2058 cells, n=54/70 vs. n=16/70 from 5 mice, Fisher’s exact test.

Next, we set out to get first insights into the dynamic regulation of the PAM pathway in brain-colonizing cancer cells at each subsequent step of the brain metastatic cascade. Hereto, A2058 melanoma cells expressing both RFP and the Akt sensor were intracardially injected in mice to perform molecular imaging by repetitive intravital multiphoton microscopy through a chronic cranial window^16^ (Figure 2A). Within two hours after injection of a population of cells with low Akt activity (due to specific *in vitro* conditions), about 77 % of tumor cells (n=54 of 70 cells in n=5 mice) arrested in brain microvessels displayed a cytoplasmic GFP signal, indicative of an activated PAM pathway (Figure 2B). After successful extravasation, all cancer cells showed high Akt activity (Figure 2B and Supplementary Figure 2). Interestingly, tracking all tumor cells over time revealed that only those cells with high Akt activity in the intravascular stage succeeded to extravasate and colonize the perivascular niche (Figure 2C, 2D). The intravascular cells with low Akt activity (23 %, n=16) were less likely to attain permanent arrest, and an extravasation event was never observed (Figure 2D). In summary, longitudinally monitoring PAM pathway activation *in vivo* demonstrated that robust and long-lasting PAM pathway activation, initiated as early as in the intravascular stage of tumor cell arrest in the brain, is critical for extravasation and subsequent melanoma BM formation.

### Heterogenous PAM pathway activation in established brain metastases

**Figure 3:**
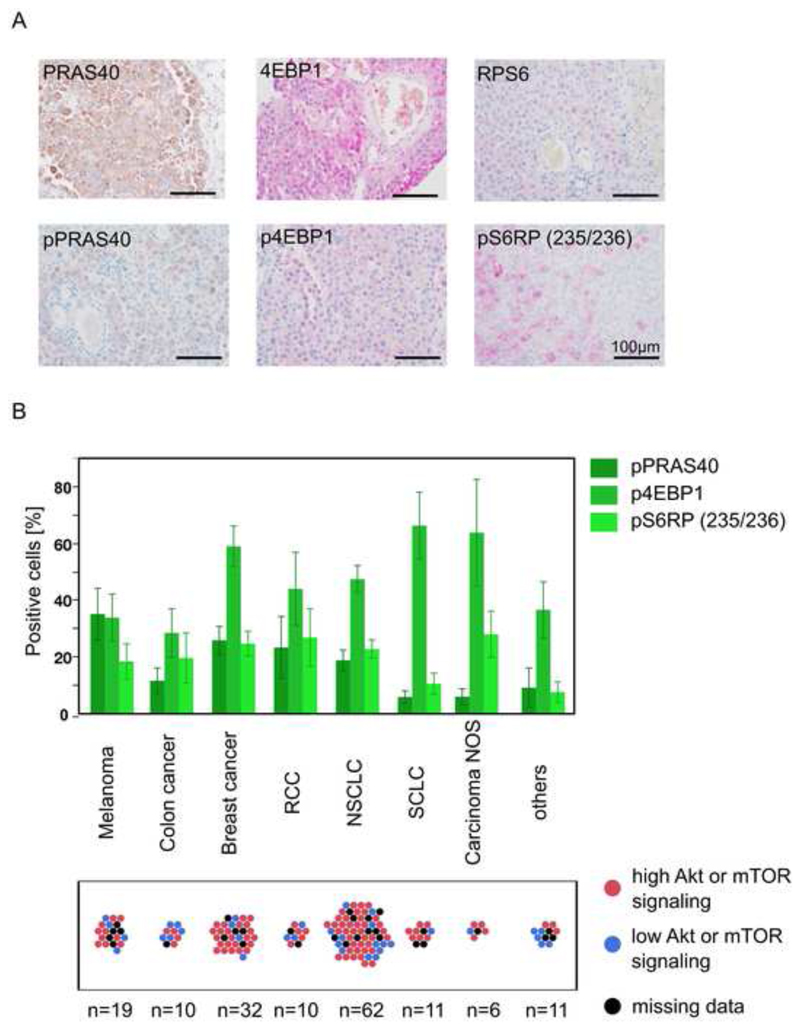
Akt and mTOR signaling in established human BM. (A) Expression of PAM pathway targets in FFPE tissue micro arrays of human melanoma BM. (B) Signaling of phosphorylated PAM pathway targets in specimen of different secondary brain tumor entities. Proportion of patients with high Akt or mTOR signaling (above median) is demonstrated. n values are indicated. Error bars show SEM.

PAM pathway activation in human BM was confirmed by analyzing tissue microarray data of patients suffering from metastatic MM (Figure 3A) and other cancers. These established human BM showed PAM pathway activation in many cases, but activation patterns were quite heterogenous (Figure 3B). Together with the biosensor study and in light of a clinical trial showing insufficient response to a PI3K inhibitor in MM patients with established BM^14^, this provided a clear rationale to experimentally test the BM-suppressive effects of GNE-317 in various schedules. Here, we hypothesize that the PAM pathway inhibitor has the strongest effects on early, fate-deciding steps of BM formation where the PAM pathway could play the most relevant and consistent role.

### Impact of dual PI3K/mTOR inhibition on melanoma cells *in vitro* and *in vivo*

**Figure 4:**
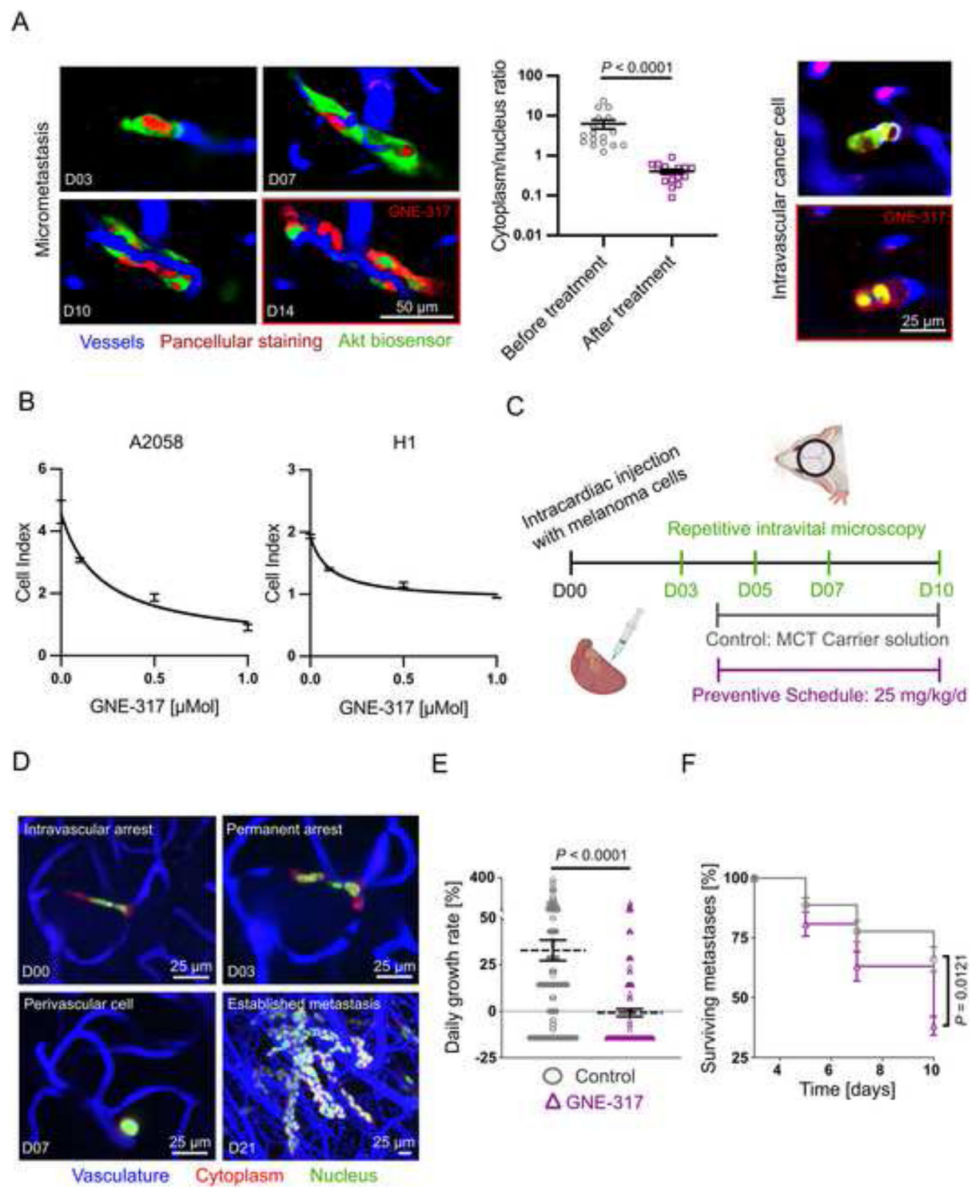
Impact of a dual PI3K/mTOR inhibitor on melanoma cells *in vitro* and *in vivo*. (A) Representative *in vivo* molecular imaging examples of biosensor labeled A2058 cells, showing a reduction of Akt activity in micrometastasis and intravascular arrested cancer cells in response to administration of GNE-317. Image quantification shows lower cytoplasm/nucleus ratios after administration of GNE-317. Intravital multiphoton microscopy, A2058 cells, n=3 mice, error bars show SEM, Mann-Whitney test. (B) Real-time proliferation assays. Dose-response curves of GNE-317 treated A2058 and H1 human melanoma cells. Error bars show SD. (C) *In vivo* treatment study with an intravital microscopy approach investigating response to early dual PI3K/mTOR inhibition. Mice in this preventive schedule group were treated with 25 mg/kg GNE-317 daily, control group with carrier solution respectively. (D) Intravital multiphoton microscopy visualizes the brain metastatic cascade. A2058 cells, maximum intensity projection. (E) Tumor growth rates per day were calculated by difference between number of cells in metastases at day 3 and day 10. A2058 cells, n=174/153 cells in n=3 mice per group, data shows mean and SEM. Mann-Whitney test. (F) Metastases survival until day 10 was investigated. Proportion of surviving metastases was calculated by numbers of remaining tumor cells in relation to initially labeled cells at day 3. A2058 cells, n=174/153 cells in n=3 mice per group, data shows mean and SEM, student’s t-test.

Thus we next performed *in vitro* and *in vivo* studies to investigate the relevance of PAM pathway inhibition for BM formation. To verify an on-target activity of the brain penetrant dual PI3K/mTOR inhibitor GNE-317^22^ *in vivo,* we performed intravital microscopy and could demonstrate a specific PAM pathway inhibition in metastasizing cancer cells in the live mouse brain (Figure 4A). Additionally, as cancer cell proliferation capacity governs macrometastasis formation, real-time cell analysis proliferation assays were performed and demonstrated dose-dependent reduction of proliferation in GNE-317 treated H1 and A2058 melanoma cells (Figure 4B), and in primary melanoma cell lines derived from human BM (Supplementary Figure 3). Also, the fraction of apoptotic cells was increased by *in vitro* treatment with GNE-317 (Supplementary Figure 4). Next, we set out to investigate whether PAM inhibition with the brain penetrant dual PI3K/mTOR inhibitor GNE-317^12^ is effective against the earliest steps of BM formation in a preclinical setting (Figure 4C). By repetitive intravital multiphoton microscopy of A2058 melanoma cells (expressing cytoplasmic RFP and nuclear GFP), proliferation and potential survival could be detected at every step of the metastatic cascade (Figure 4D)^16^. When mice were treated early on (starting day 4) with GNE-317, a significant reduction of the growth rate and also survival of individual micrometastatic lesions was found (Figure 4E, F), when compared to mock-treated controls. These data confirmed that a high, maximum-tolerated dose of GNE-317 (25 mg/kg/d) is able to exert meaningful effects on the overall brain metastatic cascade and targets melanoma tumor cells from the earliest steps of brain colonization on.

### Distinct BM-preventive effects of a PAM pathway inhibitor

**Figure 5:**
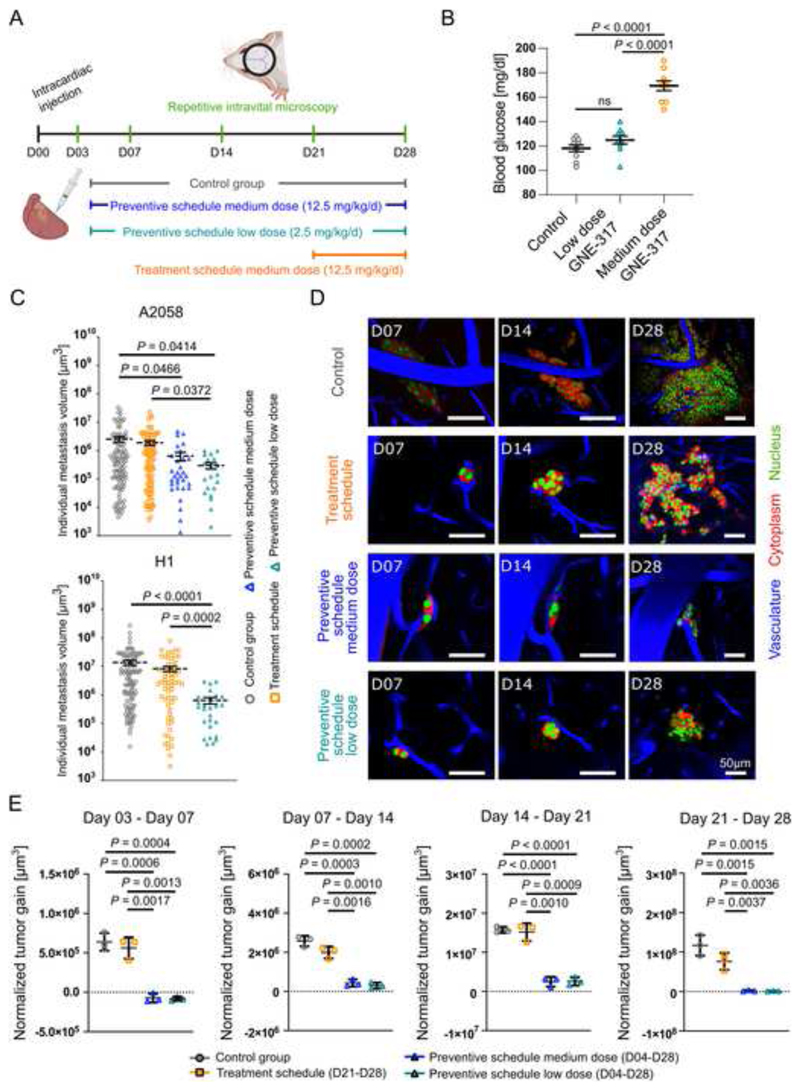
Preventive vs therapeutic schedules. (A) Longitudinal intravital multiphoton microscopy investigating GNE-317 treatment in preventive and treatment schedules in A2058 and H1 melanoma models. (B) Different doses of GNE-317 and their impact on blood glucose levels in mice intracardially injected with A2058 melanoma cells, n=10 measurements in n=5 mice. (C) Individual volumes of metastases that mastered all steps of metastatic cascade were measured at day 28. A2058 cells, intravital multiphoton microscopy, n=111/97/31/20 cells in n=3 mice per group, error bars show SEM, Mann-Whitney test. H1 melanoma cells, intravital multiphoton microscopy, n=120/64/26 cells in 3/3/4 mice per group, error bars show SEM, Mann-Whitney test. (D) Representative intravital multiphoton microscopy images visualize A2058 BM growth among groups. (E) Quantification of BM volume gain is indicated for all four groups and observation periods. Volume of BM was normalized to volume at day 3. A2058 melanoma cells, intravital multiphoton microscopy, n=179/175/205/109 cells in n=3 mice per group, error bars show SD, student’s t-test.

A potential BM-preventive drug for cancer patients needs to be administered for a long period of time, and ideally in very low doses to avoid side effects^1,6^. To this end, we investigated whether the early application of a medium and even a very low dose of GNE-317 can achieve BM prevention *in vivo* in two models of melanoma BM. Using long-term repetitive intravital multiphoton microscopy, BM development was monitored in four groups: mice receiving GNE-317 in a preventive schedule with medium (12.5 mg/kg/d) or low (2.5 mg/kg/day) doses vs. a treatment schedule in which medium dose treatment (12.5 mg/kg/d) starts when BM are already established (Figure 5A). The medium dose is in the range of doses determined in earlier toxicity and tolerability studies with this compound^12^ and comparable to the maximum tolerated doses of PAM pathway inhibitors tested in clinical trials.

Importantly, the low dose is only 20% of the medium dose, resulting in a dose reduction that would not be expected to lead to clinically relevant side effects according to published data^23,24^. We then used our mouse model to test this hypothesis. Hyperglycemia is a common clinical side effect of PAM inhibitors^23–25^. Indeed, the medium dose of GNE-317 caused significantly increased blood glucose in our mouse model compared to control (Figure 5B). In contrast, low dose GNE-317 did not show any significant effect on blood glucose levels, indicating that lowering the dose can indeed reduce crucial side effects of PAM pathway inhibitors.

At day 28 following intracardiac injection, intravital multiphoton microscopy revealed that BM size was reduced in preventive schedules in H1 and A2058 models (Figure 5C). Strikingly, both preventive schedule groups appeared to effectively arrest metastases in small micrometastatic stages (Figure 5D). Quantification of the BM growth rate over all 4 weeks of BM formation revealed that both preventive schedules indeed effectively halted tumor cell proliferation compared to the control group, with strongest effects during the first week where even temporary reductions of tumor volumes were observed (Figure 5E). Importantly, BM growth suppression achieved by the low and medium dose preventive schedule groups were comparable over the whole course of the study (Figure 5E). The strong effects of the low preventive dose were confirmed using the second H1 melanoma model (Supplementary Figure 5). In contrast to the findings with the preventive schedules, treating established metastasis with 12.5 mg/kg/d GNE-317 led only to moderate effects on the growth of established BMs (Figure 5C-E), which is in line with the lack of response of established BM when MM patients were treated with the PI3K inhibitor buparlisib^14^. These data imply that there is a substantial benefit of starting low-dose drug administration with GNE-317 during the earliest steps of brain colonization.

### Longitudinal *in vivo* imaging reveals exact drug effects

**Figure 6:**
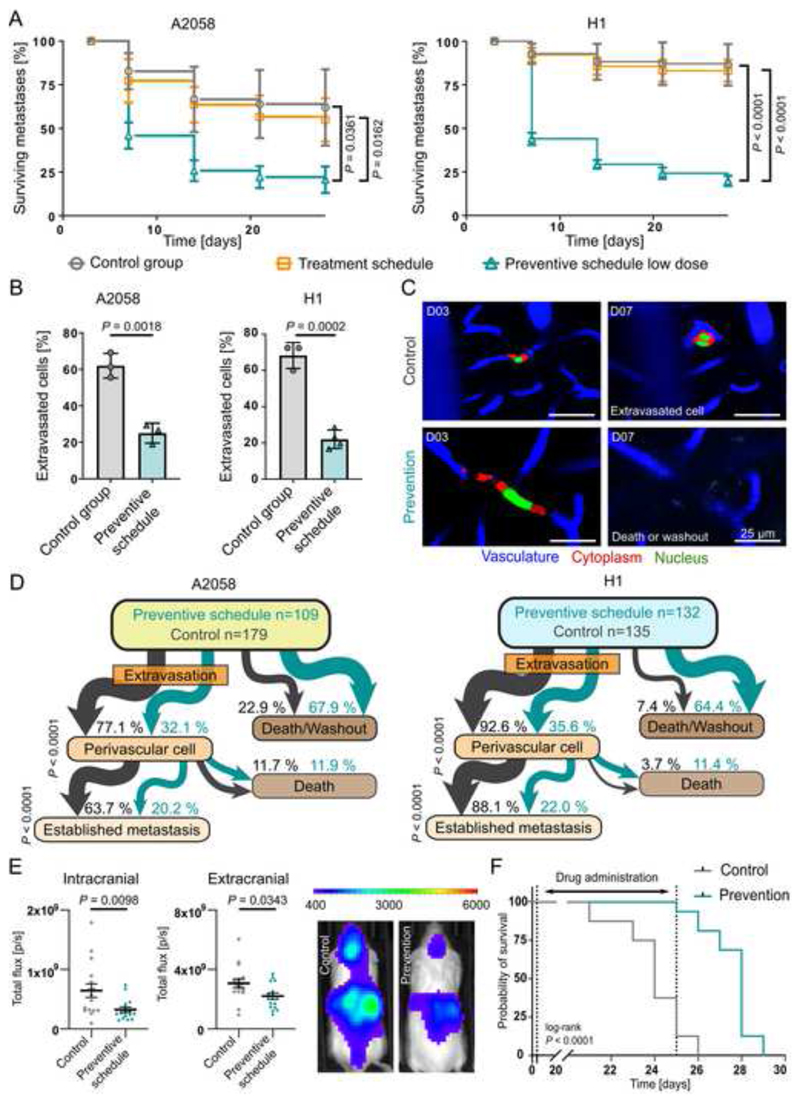
The earliest steps of the brain metastatic cascade are most vulnerable to PAM pathway inhibition. (A) Tumor cell survival between day 3 and 28 after intracardiac injection. A2058 cells, intravital multiphoton microscopy, n=179/175/205/109 cells in n=3 mice per group, error bars show SD, student’s t-test. H1 melanoma cells, intravital multiphoton microscopy, n=135/77/132 cells in 3/3/4 mice per group, error bars show SD, student’s t-test. (B) Proportion of cells that performed extravasation until day 7. A2058 cells, intravital multiphoton microscopy, n=179/175/205/109 cells in n=3 mice per group, error bars show SD, student’s t-test. H1 cells, intravital multiphoton microscopy, n= 135/77/132 cells in 3/3/4 mice per group, error bars show SD, student’s t-test (C) Representative intravital multiphoton microscopy images show brain colonization in control group and death or wash out of a tumor cell in preventive schedule group. A2058 cells. (D) Tumor cells were followed over time, and events quantified. Flow chart indicates the fate of every individual melanoma cell from day 3 to day 28. A2058 cells, intravital multiphoton microscopy, n=179/175/205/109, Fisher’s exact test. H1 cells, intravital multiphoton microscopy, n=135/77/132 cells in 3/4 mice per group, Fisher’s exact test. (E) Low dose GNE-317 administered in a preventive schedule reduced formation of intracranial and extracranial metastases of A2058 melanoma cells. Whole-body imaging (IVIS) in week 3 following intracardiac injection, n=16 mice per group, error bars show SEM, Mann-Whitney test. (F) Mice receiving low dose GNE-317 in a preventive schedule from day 4 to day 25 show increased overall survival. n=16 mice per group, log-rank test.

Next, we set out to uncover the exact step(s) of the brain metastatic cascade that are influenced by dual PI3K/mTOR inhibition. When comparing overall survival of metastatic lesions between vascular arrest at 3 days post injection to the end of the experiment, the low dose preventive schedules resulted in a reduced number of metastatic foci over time (Figure 6A). As expected, no significant difference between control and treatment group (treatment started on day 21) was observed (Figure 6A). Importantly, the strongest reduction in number of metastatic lesions by the preventive schedule was observed between day 3 and day 7 (Figure 6A). Previously, our group demonstrated that extravasation of cancer cells from the brain capillaries typically takes place in the first week following intracardiac injection^16^. Interestingly, in low dose preventive groups less melanoma cells extravasate until day 7 compared to the control group (Figure 6B, 6C). Tracking the fate of each metastatic lesion revealed that significantly less A2058 melanoma cells seeded the perivascular niche in mice under preventive treatment compared to control (32.1% vs 77.1%) and also formed less established BM (20.2% vs 63.7%) (Figure 6D). This was confirmed in the second melanoma model (Figure 6D). A similar difference was detected between the low dose prevention vs the treatment group (Supplementary Figure 6). Of note, since in the treatment group dual PI3K/mTOR inhibition only starts post-extravasation, this group can be regarded as a second independent control group for this experiment. The effect of preventive treatment therefore appears two-fold: both extravasation and also early colonization of the perivascular niche are affected by PAM pathway inhibition (Figure 6B-D). These findings were further supported with analysis of BM development among all groups in both models (Supplementary Figure 6). Importantly, there was again no significant difference in formation of established metastasis between the medium and low dose preventive schedule groups (Supplementary Figure 6).

Finally, we sought to confirm the antimetastatic effects of PAM pathway inhibition with longitudinal whole-body bioluminescence imaging and a survival study. Low dose GNE-317 in a preventive schedule significantly reduced metastatic burden inside and outside the brain, however the strongest effect was observed on intracranial metastases (Figure 6E). Furthermore, the low dose preventive schedule showed a particularly strong survival benefit (Figure 6F, Supplementary Figure 7A). Moreover, after cessation of GNE-317 administration, metastatic growth accelerated (Supplementary Figure 7B), which argues for a continues low dose administration of the drug for maximum brain metastasis suppression.

## Discussion

The data reported here collectively demonstrate that dynamic PAM pathway activation in tumor cells is a crucial first step for the brain metastatic process, and that PAM inhibition targets those rate-limiting steps of the brain metastatic cascade. This supports the notion that metastasis prevention is much easier to achieve than successful treatment of established BM disease, as the critical earliest steps of BM formation are highly vulnerable targets^6,16,26^.

We have shown that the crucial process of extravasation coincides with PAM pathway activation in the tumor cell, and can be successfully reduced by dual PI3K/mTOR inhibition. While off-target effects of GNE-317 on the melanoma cells cannot be fully excluded, the multiple well-described effects of the PAM pathway on biological functions of tumor cells^27^, but potentially also endothelial cells^28,29^, should play a central role here. However, the process of tumor cell extravasation in the brain is not well understood yet; the finding that PAM pathway is involved broadens our understanding of this crucial early and rate-limiting step of BM formation^16^. Moreover, this current study underlines the biological importance of perivascular niche colonization^30,31^ for the brain metastatic process, and provides some guidance regarding potential molecular players, too.

Prevention requires long-term administration of a drug and therefore needs to be well tolerated by patients. Clinical phase 3 trials report several side effects for alpelisib, an FDA approved PI3K inhibitor for metastatic breast cancer: hyperglycemia, diarrhea, nausea, decreased appetite and rash^23^. Moreover, treatment with buparlisib, a brain penetrant PI3K inhibitor tested in metastatic breast cancer, was associated with elevated liver enzymes, hyperglycemia, nausea, diarrhea, fatigue and particularly depression^32^. Both inhibitors showed reduced side effects when lower doses were administered^24,25,33^. Importantly, alpelisib phase 1b clinical trials demonstrate decrease in side effects in reduced dosage (total adverse events grade ≥3 in 83,3% (300 mg alpelisib + paclitaxel) vs 33,3% (150 mg alpelisib + paclitaxel) of patients)^25^. Dose reduction of 25% led to disappearance of dose limiting toxic effects^24^. Likewise, the tolerability of the brain penetrant mTOR inhibitor everolimus was dose-dependent, and the drug was well tolerated in 4-fold decreased maximum tolerated dose^34^. In our preclinical studies, we demonstrated an equal effectiveness of PI3K inhibition against BM formation in preventive schedules of medium and low doses. This suggests that for the class of PAM pathway inhibitors, a low dose tertiary preventive schedule can effectively prevent BM with a favorable safety profile.

The results of this preclinical study underscore the rationale and effectivity of targeted therapies for BM prevention, a concept that is also supported by recent data of decreased BM development in lung cancer patients receiving the ALK inhibitor alectinib^35^. In order to select those patients that would benefit most from such preventive treatment, PAM pathway upregulation in circulating cancer cells would be an interesting biomarker worth of further exploration^36,37,38^.

In conclusion, the effectivity of a brain penetrant dual PI3K/mTOR inhibitor in low doses particularly on the prevention of brain metastases makes this approach to a plausible road for a future BM prevention trial in high-risk MM patients. Until today, only one clinical trial is active that explicitly tests prevention of BM by testing the chemotherapeutic brain-penetrant drug temozolomide in breast cancer patients (NCT03190967)^39^. Effective BM prevention by a well-tolerated drug that can be administered over prolonged periods of time could make a real difference for cancer patients in the future.

## Supplementary Material

Supplementary Figure 1

## Key points

1. PI3K/mTOR inhibition is effective in *preventing* brain metastases formation
2. Low dose PI3K/mTOR inhibition emerges as a novel antimetastatic strategy for MM
3. Molecular imaging identifies PAM pathway activation as crucial for brain colonization

## Importance of the study

Despite improvement of systemic therapies, patients with brain metastases still face a poor prognosis. Somatic genetic alterations in the PI3K pathway often occur in brain metastases, and inhibition of the PI3K/Akt/mTOR pathway has been clinically investigated. However, side effects of this drug class can be severe and led to dose-dependent adverse events-related discontinuation rate of as many as 25 % in phase 3 trials. Administering similar drugs in a preventive schedule might be an opportunity to lower their dose, making their administration clinically feasible, and at the same time effectively prevent the earliest steps of brain colonization. The results presented here indicate that low-dose dual PI3K/mTOR inhibition over prolonged periods of time is indeed a promising strategy for brain metastasis prevention in high-risk malignant melanoma patients. Clinical trials are needed to further evaluate this novel approach for clinically feasible brain metastasis prevention.
