# Supplementary material for: The PI3K/Akt/mTOR pathway as a preventive target in melanoma brain metastasis
Source: Neuro Oncol. Author manuscript; Available in PMC 2022 Feb 2. (PMC8804893; doi:10.1093/neuonc/noab159)
Supplement: Supplementary Figure 1 [file EMS131034-supplement-Supplementary_Figure_1.pdf]

Supplementary Figures and Figure Legends (revised)

Click here to access/download;Supplementary file;Supplementary Figures and Figure

# Supplementary Figure 1

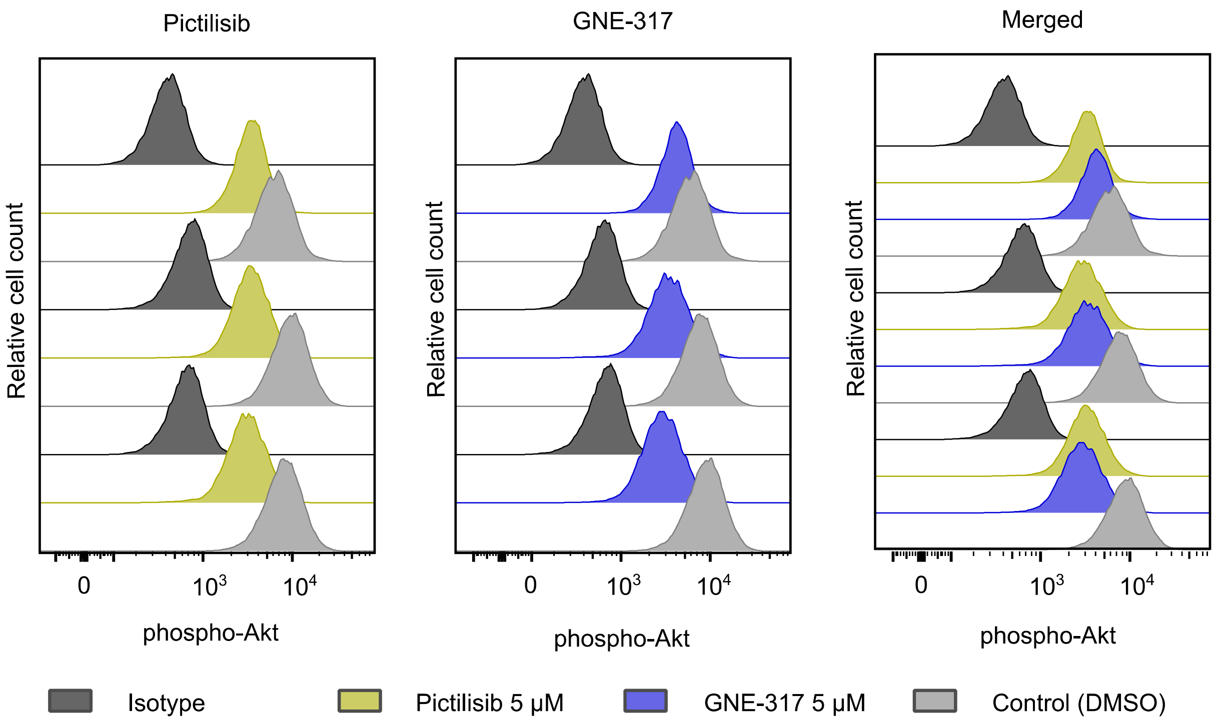


**Supplementary Fig. 1:**

GNE-317 and pictilisib reduce levels of phosphorylated Akt. Flow cytometry analysis demonstrate decrease in phosphorylated Akt levels in GNE-317 or pictilisib treated cells. Treatment was administered with a concentration of 5 μM or same amount of DMSO (control) for 10h. A2058 cells, n=3 independent experiments.

# Supplementary Figure 2

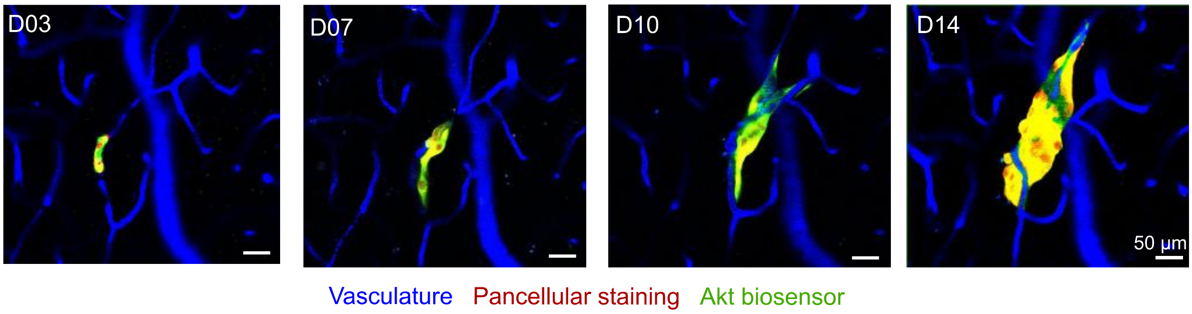


**Supplementary Fig. 2:**

Molecular imaging reveals constant high Akt activity of extravasated tumor cells. Longitudinal following up on a melanoma cell during process of BM formation. An intravascular melanoma cell with high Akt activity extravasates and tumor cells show constant PAM pathway activation once they colonized the brain. Intravital multiphoton microscopy, A2058 cells, green: biosensor, red: pancellular staining, blue: brain vessels.

# Supplementary Figure 3

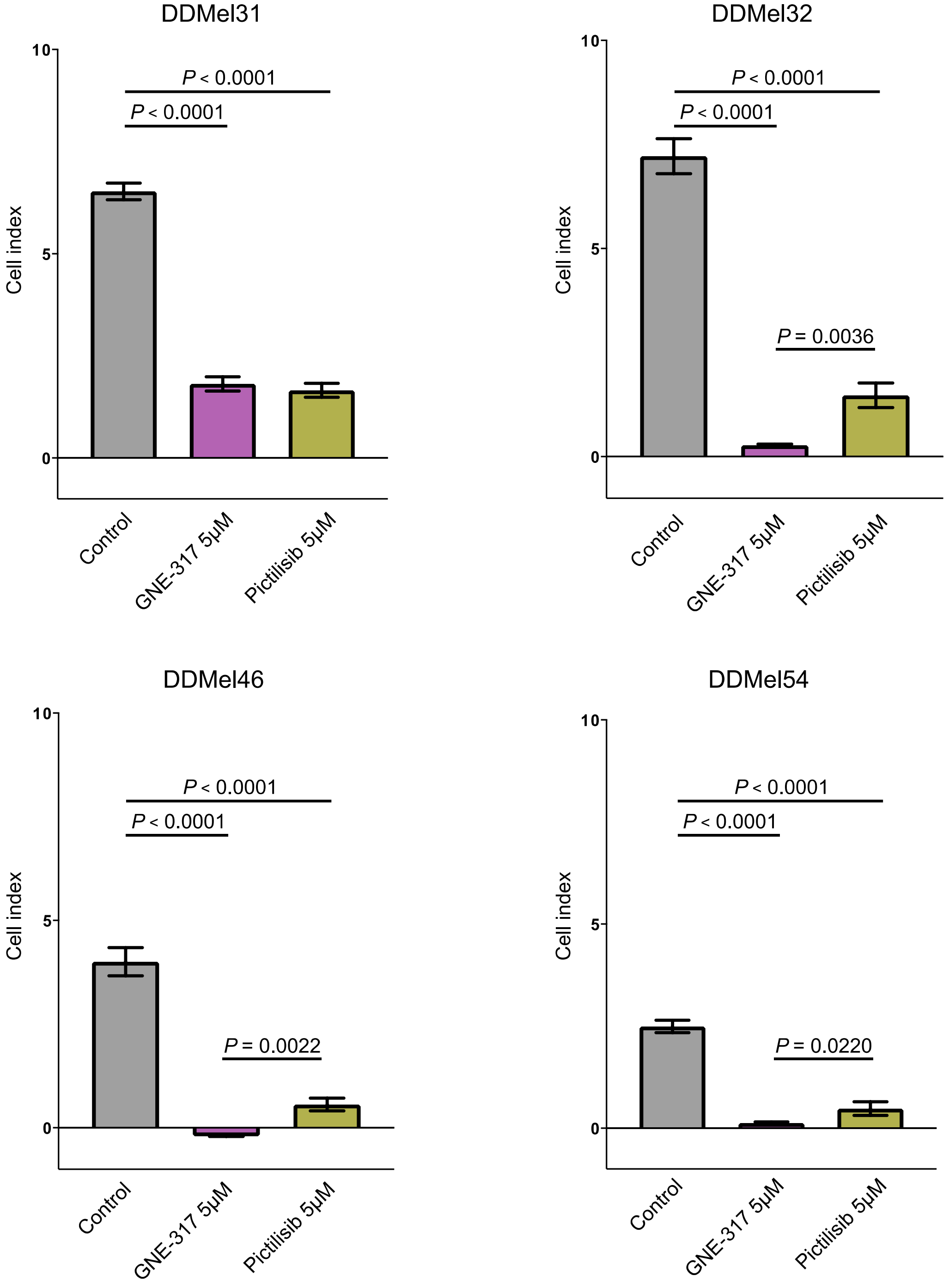


**Supplementary Fig. 3:**

Inhibition of PAM pathway reduces proliferation of primary melanoma cell lines *in vitro.* Real-time cell analyzer assays show anti-proliferative effect of dual PI3K/mTOR inhibitor GNE-317 and selective PI3K inhibitor pictilisib in primary melanoma cell lines DDMel31, 32, 46 and 54, n=8/4 replicates per concentration, error bars show SD, student's t-test.

# Supplementary Figure 4

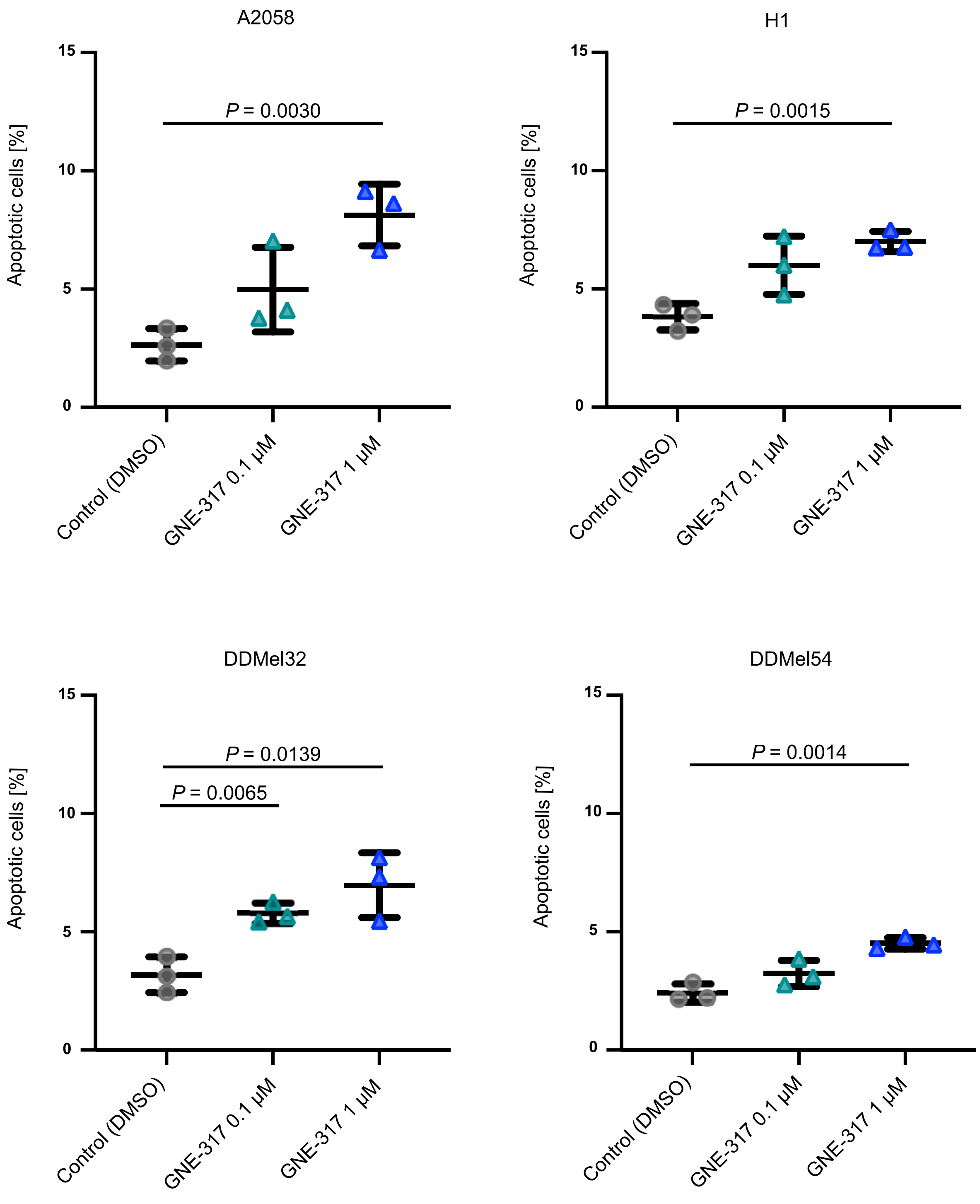


**Supplementary Fig. 4:**

Inhibition of PAM pathway induces apoptosis *in vitro.* Flow cytometry analysis demonstrates increase of early and late apoptotic cells in melanoma cells treated with GNE-317 or same amount of DMSO (control). A2058, H1 and primary melanoma cells DDMel32 and DDMel54, n=3 independent experiments, student's t-test, error bars show SD.

# Supplementary Figure 5

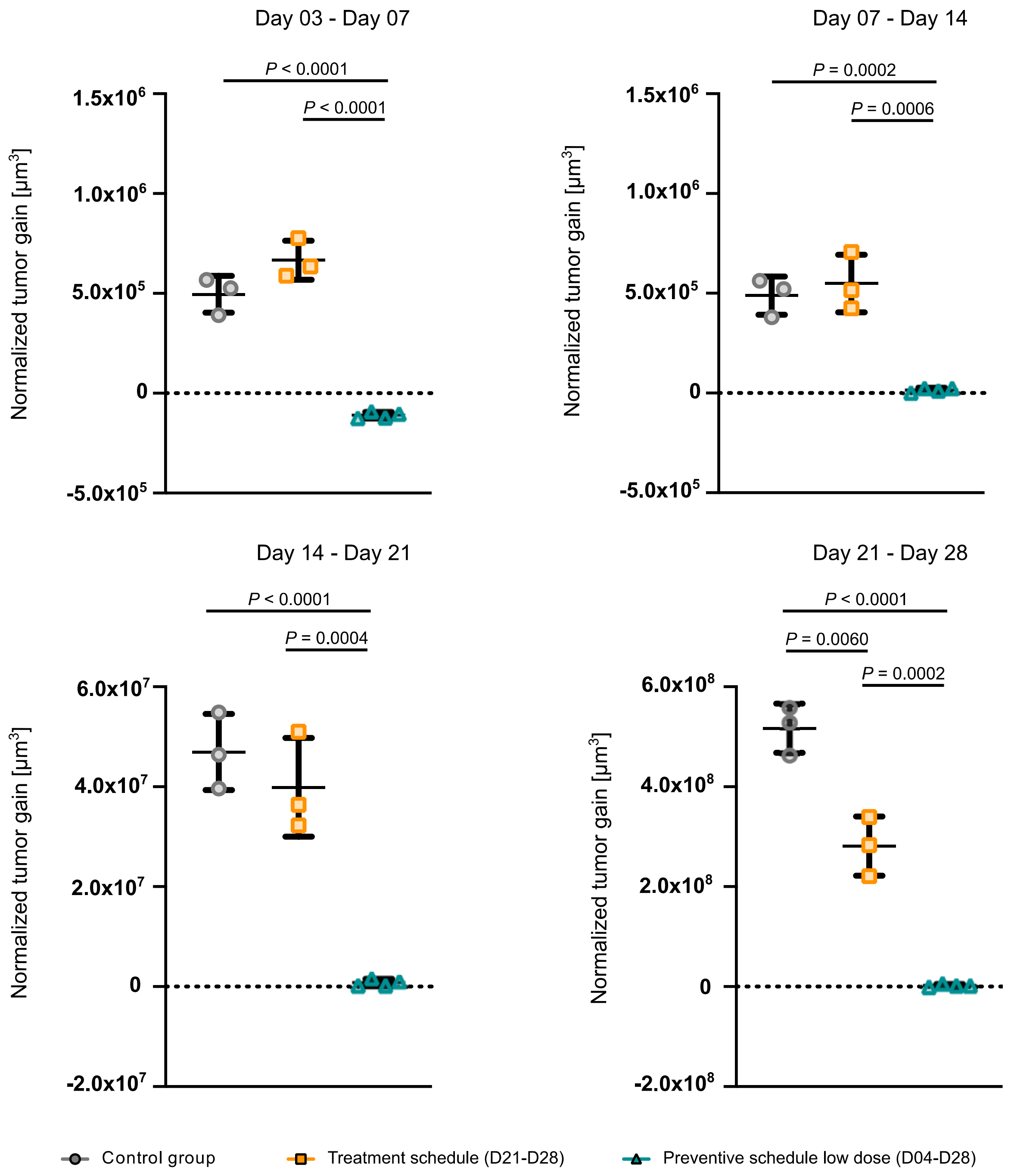


**Supplementary Fig. 5:**

Low dose preventive schedule successfully reduces brain metastasis burden in H1 melanoma model. Tumor gain were calculated for all observation periods. Volume of brain metastasis was normalized to volume at day 3. H1 cells, intravital multiphoton microscopy, n=135/77/132 cells in 3/3/4 mice per group, error bars show SD, student's t-test.

# Supplementary Figure 6

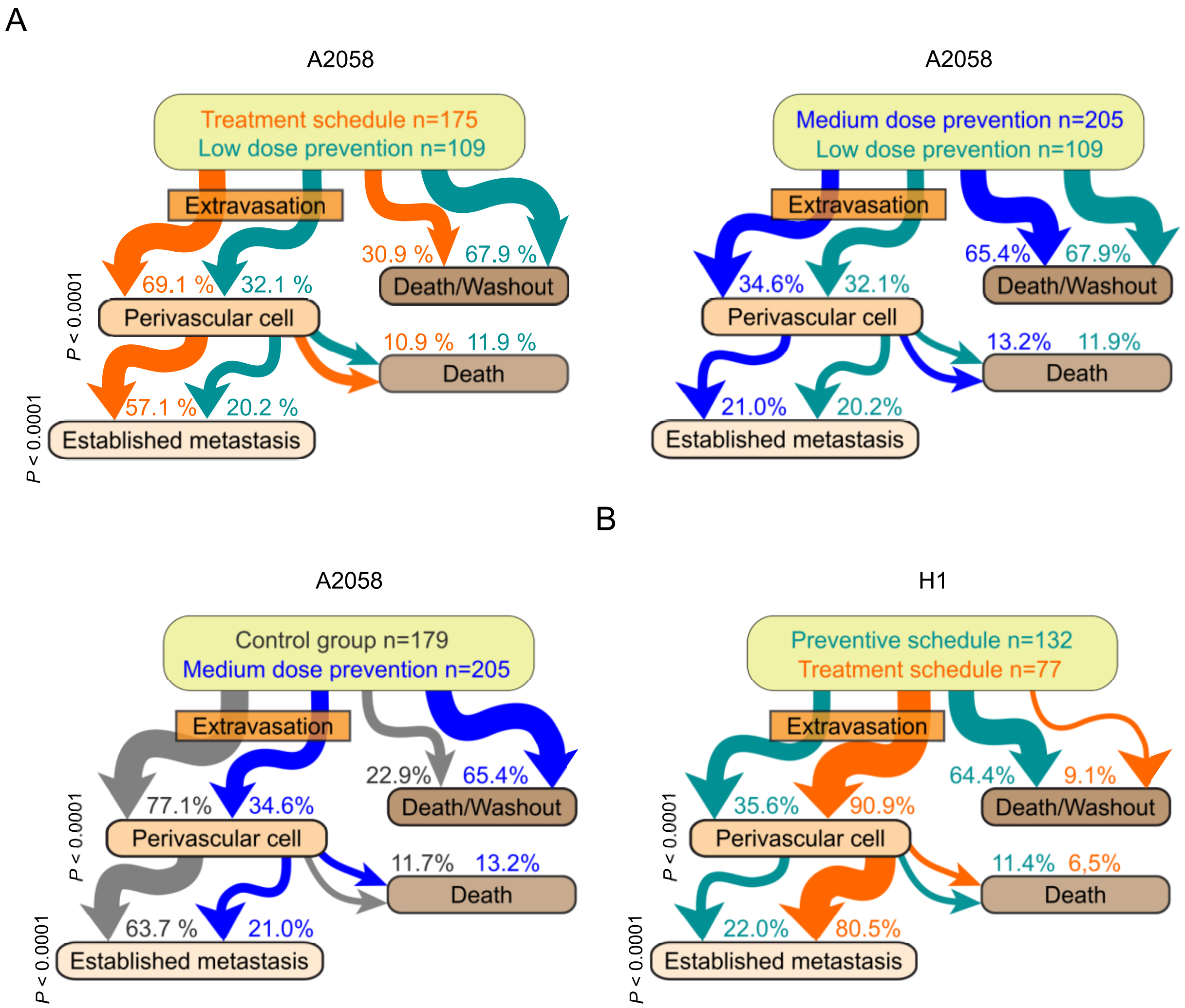


**Supplementary Fig. 6:**

Early PAM pathway inhibition prevents outgrowth of established metastasis. (A and B) Flow charts indicates development of metastasis among different groups. Values refer to initial detected cells at day 3. (A) A2058 melanoma cells, intravital multiphoton microscopy, n=179/175/205/109, Fisher's exact test. (B) H1 cells, intravital multiphoton microscopy, n= 135/77/132 cells in 3/3/4 mice per group, Fisher's exact test.

# Supplementary Figure 7

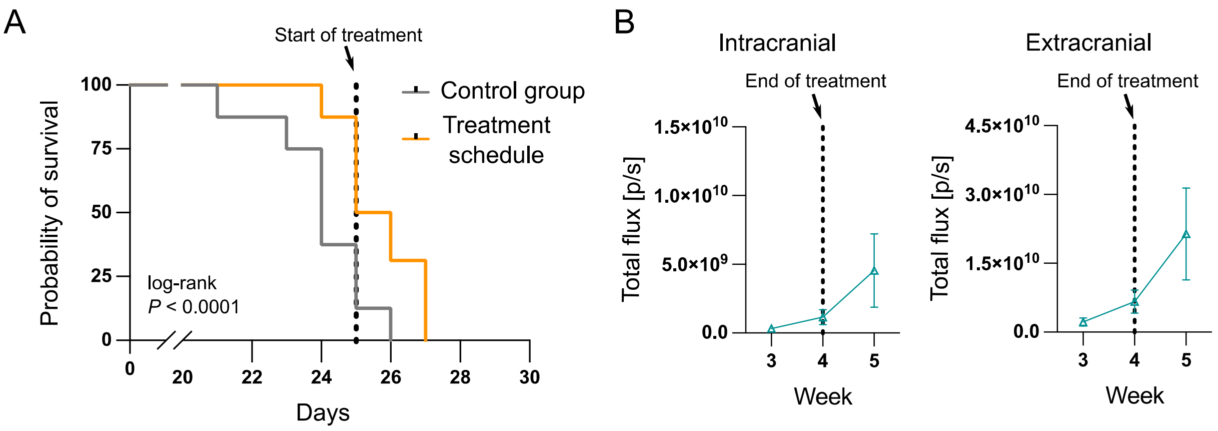


**Supplementary Fig. 7:**

Effect of GNE-317 on tumor growth and overall survival. (A) Mice receiving medium dose GNE-317 in a treatment schedule with oral gavage from day 25 on showed only moderately prolonged overall survival compared to the control group receiving carrier solution via oral gavage from day 4 to 25. Note that less distressing interventions (oral gavages) occurred in the treatment schedule group which might lead to an overestimation of the survival benefit by GNE-317 treatment. n=16 mice per group, log-rank test. (B) Whole-body bioluminescence imaging (IVIS) was used to monitor intra- and extracranial metastatic burden in the preventive schedule group during week 3, 4 and 5. The findings demonstrate an acceleration of metastatic tumor growth after the preventive administration of GNE-317 was stopped (week 4). IVIS, n=16 mice, error bars show SD.

Click here to access/download;Supplementary file;Tehranian_SupplementaryMethods_clean_copy.docx

## Supplementary Material and Methods

### Cell lines and culturing conditions

The primary cell lines DDMel31 (BRAF-V600E-, NRASQ61+), DDMel32 (BRAF-V600E-, NRASQ61R+), DDMel46 (BRAF-V600E+, NRAS-) and DDMel54 (BRAF-V600E-, NRAS-) were isolated from human melanoma BM (Dresden, Germany). In order to exclude the possibility of establishing tumor associated fibroblasts, the primary cell lines were tested for their NRAS or BRAF mutation status and for their expression of the melanoma markers Melan A, tyrosinase and HMB45. These cells were cultured in RPMI medium with 10% fetal bovine serum, 1% penicillin-streptomycin-amphotericin B and 1% L-glutamine. The human melanoma cell line H1DI2 (BRAF-V600E+/-, PTEN+/) was isolated from a melanoma BM[1], and for human melanoma A2058 cells (BRAF-V600E+/-, PTEN+/-, RB1+/-, p53-/-) brain-passaging was performed[2]. The cells were cultured in DMEM, 10% fetal bovine serum and 1% penicillin-streptomycin-amphotericin B. They were regularly checked for mycoplasma infections by PCR and authenticated using multiplex human cell line authentication test. For *in vivo* imaging cells expressing green fluorescent protein (GFP) or red fluorescent protein (RFP) were used[3].

### Flow cytometry

Following control or PAM pathway inhibition of A2058 cells, apoptosis was detected by staining with annexin V-APC (1:100; BioVision) in Annexin V binding buffer (eBioscience) and fixable viability dye eFluor780 (eBioscience). Annexin V single positive cells were defined as early apoptotic, while double stained cells were defined as late apoptotic. For intracellular staining, cells were fixed and permeabilized with intracellular fixation and permeabilization buffer set (eBioscience). After blocking with human serum (1:10) for 20 min on ice, intracellular staining of APC-conjugated rabbit anti-phospho-Akt (1:50; #11962; Cell Signaling) or isotype control (#12445, CellSignaling) was performed for 45 min.

### Western blot

Following PAM pathway inhibition or control treatment, cells were lysed with ice-cold RIPA buffer. RIPA buffer consists of 50 mM Tris, pH 7.4 (A1086,1000, AppliChem Panreac), 150 mM NaCl (31434-1KG, Sigma), 1% Triton X-100 (A4975, AppliChem Panreac), 0.5% sodium deoxycholate (A1531-0100, AppliChem Panreac), 1 mM EDTA (AM9260G, Thermo Fisher Scientific), 1 mM EGTA

(15425795, Alfa Aesar), 0.5 mM PMSF (6367.2, Roth), Complete protease inhibitor (4693132001, Roche) and HALT Phosphatase inhibitor (78420, Thermo Fisher Scientific). Protein concentrations were measured using the BCA kit (23225, Thermo Fisher Scientific). 7.5 µg protein diluted with NuPAGE LDS 4x sample buffer (NP007, Thermo Fisher Scientific) and NuPAGE 10x sample reducing agent (NP004, Thermo Fisher Scientific) was denatured and electrophoretically separated on NuPAGE 4-12% Bis-Tris Mini Gel (NP0321BOX, Thermo Fisher Scientific) in Novex™ NuPAGE™ MOPS SDS running buffer (NP001, Thermo Fisher Scientific) with NuPAGE Antioxidant (NP005, Thermo Fisher Scientific). Proteins were blotted onto a PVDF membrane (IPVH00010, Merck) and blocked with 5% albumin (8076.4, Roth) or milk powder (T145.2, Roth) in 5 mM Tris (A1086,1000, AppliChem Panreac), 16 mM Tris-HCl (9090.3, Roth), 150 mM NaCl (3134-1KG-M, Sigma), 0.15% Tween 20 (500-018-3. MPBio) for 1 h. Primary antibodies against Akt (4685), p-Akt(S473) (4060), S6RP (2317), p-S6RP (S235/236) (2211), mTOR (2983), p-mTOR (S2448) (2971), and loading control mouse anti- β-tubulin (2146) were used (all: 1:1000, Cell Signaling). Chemiluminescent development was done using Clarity Western ECL Substrate (170-5060, Bio-Rad).

For stripping, the membrane was incubated for two hours in a stripping buffer containing 0.15% glycine (33226-1kg, Sigma) 0.1% SDS (A1112,1000, Applichem PanReac, 1% Tween-20 (500-018-3. MPBio), pH 2.2 and rinsed two times with PBS composed of 137 mM NaCl (31434-1KG, Sigma), 2.7 mM KCl (6781.1, Roth), 10 mM Na2HPO4 (P030.3, Roth), 1.8 mM KH2PO4 (3904.1, Roth) and TBS-T respectively. Raw images were processed with Image Lab Software (Bio-Rad). Windows Photos (Microsoft) was used for rotating the chemilumincescent images and modulating the light intensity.

**Immunohistochemistry**

Immunohistochemistry was performed using the automated IHC staining system Discovery XT (Roche/Ventana). In contrast to the described immunohistochemistry protocol[4], we used the Discovery RedMap Kit (Roche/Ventana) for detection, which includes the enzyme alkaline phosphatase and the substrate Fast Red for visualization of RPS6 full protein, 4EBP1 full protein, phospho-RPS6 (Ser235/236) as well as phospho-4EBP1 (Thr37/46). For detection of PRAS40 full protein and phospho-PRAS40 we used a standard protocol with the DAB Map Kit (Roche/Ventana). The following antibodies were used (Cell Signaling): phospho-S6RP (Ser235/236) (1:400; D57.2.2.E); phospho-4EBP1 (Thr37/46) (1:1000; 236B4), phospho-PRAS40 (1:200; D4D2), S6RP (1:100; 5G10), 4EBP1

(1:400; 53H11) and PRAS40 (1:200; D23C7). Scoring was performed by two investigators (MWR and PNH). Positive signals in tumor cells as percentage of the whole amount of tumor cells were scored.

**Proliferation assays *in vitro***

Cells were seeded in duplets/ quadruplets/ octuplets of 2,000 cells each in an RTCA E-plate and treated with different doses of dual PI3K/mTOR inhibitor GNE-317[5] or the selective pan-class I PI3K inhibitor Pictisilib[6] (0.1/0.5/1/10/30 µmol/l) in 0.5% DMSO and culture media. Cells were observed until cell density reached a plateau, and the maximum cell index during this time was used for the dose–response analysis with the RTCA Software 1.2.1.1002 (ACEA Biosciences)[7].

**Intravital multiphoton laser scanning microscopy**

The ZEISS LSM 7MP is equipped with a 20x/1.0 W-Plan-Apochromat objective (ZEISS) and a BP500-550 / BP575-610 Filter (ZEISS). Images were acquired using lowest adequate laser power and a gain ranging from 600 to 800. Z-steps were 1 or 3 µm, depending on the experiment (biosensor studies: 1µm, volume measurements: 3 µm). Tail vein injection of 100 µl of tetramethylrhodamine-isothiocyanate-Dextran (5 mg m, TRITC Dextran; average molecular weight: 500,000, Sigma-Aldrich) was performed to visualize vasculature *in vivo*. Used excitation wavelength was dependent on the cell's fluorophore: 850, 900 or 950 nm (for visualization of GFP and TRITC-Dextran, Akt sensor, and tdTomato respectively). The mice were anesthetized by applying isofluran through a nose mask. After being adequately anesthetized, mice were painlessly fixated in a custom-made holder. To be certain to image the same cells over time, correlation between stereotactic coordinates of the microscope and both local and superficial brain vasculature architecture was performed at every imaging time point.

**Akt biosensor studies**

To visualize Akt activity of intracardially injected A2058 melanoma cells *in vivo*, a transduction with Akt biosensor and a pancellular RFP-marker was performed. Due to *in vitro* growth conditions and preparation for intracardiac injection, A2058 cells displayed low Akt activity at the time of the injection[8]. Malignant melanoma cells were seeded in a low confluency. Medium was removed, cells were washed with phosphate buffered saline and medium was later replaced with serum-free medium, as described before[8–10]. Preparation of the cells for intracardiac injection was performed under serum-free conditions.

**Whole-body imaging and survival study**

*In vivo* bioluminescence imaging (IVIS Lumina Series III Imaging system, PerkinElmer) was performed to measure intra- and extracranial metastases. D-Luciferin (150 mg/kg, BioVision) was injected intraperitoneally. Mice were sedated with isoflurane and IVIS imaging was performed 10 minutes after luciferin injection. Images were acquired using the XFOV- 24 lens and the auto exposure function (medium binning, 1.2 F/Stop). Quantification and image processing was performed with Living Imaging Software (PerkinElmer). Regions of interest were defined for each mouse (intracranial/extracranial) and total flux was quantified. The smoothing function (3x3) of Living Imaging software was used for image processing of representative examples. Clinical scoring was performed daily, and mice were euthanized as soon as humane endpoint was reached (in accordance with animal regulations).

***In vivo* microscopy data analysis and quantification**

Initial image processing and quantification was performed in ZEN (Carl Zeiss Jena, Germany) and ImageJ[11]. Image adjustment was applied to the whole image. In 3D projections, volume was measured, and nuclei were counted at each time point using the surface and split function of Imaris. Afterwards, growth rates and tumor volume gain were calculated. Cells arrested in brain vasculature during 24 hours after intracardiac injection were grouped as “intravascular arrested cells”. When cells remained in the vessel for more than 24 hours, it was counted as an event of permanent arrest. As previously reported, micrometastases were defined as metastases with 3 to 50 cells, and metastases with >50 cells or equivalent volume were called macrometastases[3]. Primary image calculation (e.g., subtraction of different channels to remove unspecific background or filtering with a median filter) was performed with ImageJ. Single planes and maximum intensity projections (MIP) were used for visualization.

**References**

1. Wang J, Daphu I, Pedersen PH, et al. A novel brain metastases model developed in immunodeficient rats closely mimics the growth of metastatic brain tumours in patients. *Neuropathol Appl Neurobiol*. 2011;37(2):189-205. doi:10.1111/j.1365-2990.2010.01119.x
2. Valiente M, Van Swearingen AED, Anders CK, et al. Brain Metastasis Cell Lines Panel: A Public Resource of Organotropic Cell Lines. *Cancer Res*. 2020;80(20):4314-4323.

doi:10.1158/0008-5472.can-20-0291

3. Osswald M, Blaes J, Liao Y, et al. Impact of blood-brain barrier integrity on tumor growth and therapy response in brain metastases. *Clin Cancer Res*. 2016;22(24):6078-6087. doi:10.1158/1078-0432.CCR-16-1327

4. Harter PN, Jennewein L, Baumgarten P, et al. Immunohistochemical assessment of phosphorylated mTORC1-pathway proteins in human brain tumors. *PLoS One*. 2015;10(5):1-20. doi:10.1371/journal.pone.0127123

5. Salphati L, Heffron TP, Alicke B, et al. Targeting the PI3K pathway in the brain - Efficacy of a PI3K inhibitor optimized to cross the blood-brain barrier. *Clin Cancer Res*. 2012;18(22):6239-6248. doi:10.1158/1078-0432.CCR-12-0720

6. Folkes AJ, Ahmadi K, Alderton WK, et al. The identification of 2-(1H-indazol-4-yl)-6-(4-methanesulfonyl-piperazin-1- ylmethyl)-4-morpholin-4-yl-thieno[3,2-d]pyrimidine (GDC-0941) as a potent, selective, orally bioavailable inhibitor of class I PI3 kinase for the treatment of cancer. *J Med Chem*. 2008;51(18):5522-5532. doi:10.1021/jm800295d

7. Stefanowicz-Hajduk J, Ochocka JR. Real-time cell analysis system in cytotoxicity applications: Usefulness and comparison with tetrazolium salt assays. *Toxicol Reports*. 2020;7(October 2019):335-344. doi:10.1016/j.toxrep.2020.02.002

8. Gross SM, Rotwein P. Akt signaling dynamics in individual cells. *J Cell Sci*. 2015;128(14):2509-2519. doi:10.1242/jcs.168773

9. Gross SM, Dane MA, Bucher E, Heiser LM. Individual Cells Can Resolve Variations in Stimulus Intensity along the IGF-PI3K-AKT Signaling Axis. *Cell Syst*. 2019;9(6):580-588.e4. doi:10.1016/j.cels.2019.11.005

10. Gross SM, Rotwein P. Quantification of growth factor signaling and pathway cross talk by live-cell imaging. *Am J Physiol - Cell Physiol*. 2017;312(3):C328-C340. doi:10.1152/ajpcell.00312.2016

11. Schindelin J, Arganda-Carreras I, Frise E, et al. Fiji: An open-source platform for biological-image analysis. *Nat Methods*. 2012;9(7):676-682. doi:10.1038/nmeth.2019
